# Data–Physics Fusion-Driven Defect Predictions for Titanium Alloy Casing Using Neural Network

**DOI:** 10.3390/ma17102226

**Published:** 2024-05-09

**Authors:** Peng Yu, Xiaoyuan Ji, Tao Sun, Wenhao Zhou, Wen Li, Qian Xu, Xiwang Qie, Yajun Yin, Xu Shen, Jianxin Zhou

**Affiliations:** 1 School of Materials Science and Engineering, State Key Laboratory of Materials Processing and Die & Mould Technology, Huazhong University of Science and Technology, Wuhan 430074, China; d202280419@hust.edu.cn (P.Y.); m202371067@hust.edu.cn (T.S.); zwhicpns@163.com (W.Z.); liwenhust@hust.edu.cn (W.L.); 22113042@whpu.edu.cn (Q.X.); qiexiwang@163.com (X.Q.); yinyajun436@hust.edu.cn (Y.Y.); shenxu2006@163.com (X.S.); zhoujianxin@hust.edu.cn (J.Z.); 2 Aeronautical Materials Research Institute, Beijing 100094, China

**Keywords:** investment casting, shrinkage defects, Ti alloy, multi-regression, neural network

## Abstract

The quality of Ti alloy casing is crucial for the safe and stable operation of aero engines. However, the fluctuation of key process parameters during the investment casting process of titanium alloy casings has a significant influence on the volume and number of porosity defects, and this influence cannot be effectively suppressed at present. Therefore, this paper proposes a strategy to control the influence of process parameters on shrinkage volume and number. This study constructed multiple regression prediction models and neural network prediction models of porosity volume and number for a ZTC4 casing by simulating the gravity investment casting process. The results show that the multiple regression prediction model and neural network prediction model of shrinkage cavity total volume have an accuracy of over 99%. The accuracy of the neural network prediction model is higher than that of the multiple regression model, and the neural network model realizes the accurate prediction of shrinkage defect volume and defect number through pouring temperature, pouring time, and mold shell temperature. The sensitivity degree of casing defects to key process parameters, from high to low, is as follows: pouring temperature, pouring time, and mold temperature. Further optimizing the key process parameter window reduces the influence of process parameter fluctuation on the volume and number of porosity defects in casing castings. This study provides a reference for actual production control process parameters to reduce shrinkage cavity and loose defects.

## 1. Introduction

ZTC4(Ti-6Al-4V) is a kind of *α-β* casing for Ti alloys, which is widely used in the manufacture of complex aviation structural parts because of its good casting and corrosion resistance [1,2,3,4,5]. In order to meet the Ti alloy casings requirements of complex functionalization, thin walls, and being lightweight, investment casting technology has been used [6,7,8]. However, the investment casting will inevitably produce shrinkage cavity [9] and loose [10] defects, which will have a negative impact on the performance of the castings. In order to minimize the shrinkage cavity and loose defects, the investment casting process parameters need to be controlled.

In the Ti alloy casing complex precision casting process, there are many urgent problems to solve. The large range of size changes and the different thicknesses of the regional organization in the solidification process is often difficult to coordinate. During the subsequent stages of hot isostatic pressing [11], weld repair [12], and heat treatment [13], different shrinkage cavity and loose defects in the same type of castings lead to different casting properties. In addition, the sequence between the inside and outside of the casting during solidification is different. The isolated liquid phase regions that solidify last are prone to the formation of shrinkage cavity and loose defects. When larger shrinkage cavities and loose defects are present, even after a hot isostatic pressing treatment, it remains challenging to eliminate the impact of these defects on the performance of casting. In order to reduce the defects of castings, many researchers have carried out corresponding studies. Yu [14] developed a numerical simulation software for the solidification process of investment casting steel, which can be used for defect prediction. Huang [15] used simulations to optimize the investment casting parameters for toothed chain joints made of SNCM220 alloy and reduced harmful defects. Pan [16] established a simulated macro-scale and micro-scale comprehensive model to achieve an accurate prediction of the microstructure of nickel-based alloy investment castings. Nan [17] improved the inner runner and the straight runner, through simulations, to ensure the casting quality and production efficiency.

Due to its large scale, numerical simulation takes a long time. It is difficult for managers to better understand the influence of parameter fluctuations on product quality. With the rapid development of neural networks, the effect of process parameter fluctuations on complex casting defects can be effectively investigated. Sata [18] proposed a prediction penalty index (ppi) and compared it to the relative predictive capability of neural and multivariate regression models, and found that multiple regression was better at predictions. The CNN (convolutional neural networks) model used by Dong [19] can accurately predict the shrinkage deformation and trend of complex castings during precision casting. Yu [20] proposed a data-driven framework, which improved the yield of castings by 14.91% by optimizing the process parameters. Jin [21] proposed the Bayesian network and an industrial computerized tomography method to improve the dimensional accuracy of casting in the IC (investment casting) process. Tian [22] designed a series of steel castings, and through the known geometric parameters of the computer-aided casting design model, the shrinkage rate could be well predicted. Manjunath [23] established a neural network model that could effectively predict casting density, hardness, and secondary dendrite arm spacing, in both forward and reverse mappings. In this paper, the data set is constructed using a physical model, and the casting defects were predicted using multiple regression and a neural network.

In order to reduce the shrinkage cavity and loose defects, we must optimize the process parameter ranges. Finding the specific range using only numerical simulation requires a long calculation time, which is not conducive to finding the optimal process range quickly. After simulating part of the experimental data through a physical model, a neural network model is constructed using the data. The ranges of the process parameters can be quickly found through the weighted shrinkage cavity and loose defect evaluation system. It is of great significance to determine the range of production parameters for complex castings.

## 2. Materials and Methods

### 2.1. Data–Physics Fusion-Driven Framework

**Figure 1:**
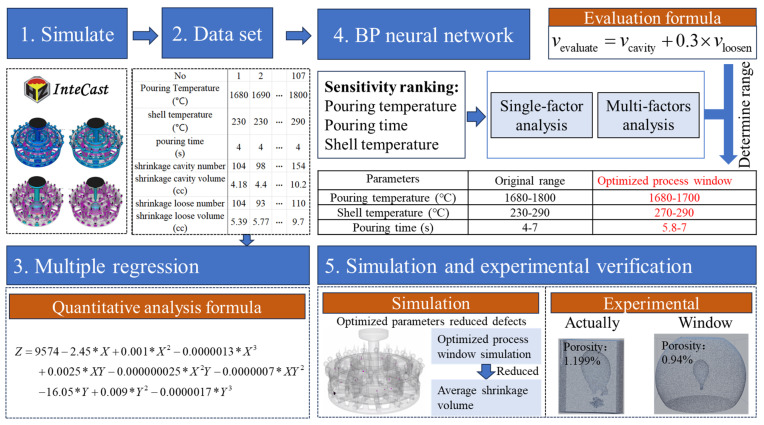
Major steps of data–physics fusion-driven framework.

In this work, the relationship between pouring temperature, mold shell temperature, pouring time, and shrinkage cavity and loose defects of castings was explored. Multiple regression [24,25] and BP (back propagation, BP) neural network [26,27,28] models were constructed to predict the influence of process parameters on the defects, by combining simulation and neural network, as shown in Figure 1. Firstly, this study uses InteCast CAE (computer-aided engineering) for the numerical simulation of complex casting. Secondly, based on the simulation results, a multiple regression analysis of the quantitative relationship between the shrinkage cavity and loose defect number and volume and the casting temperature, shell temperature, and pouring time was constructed. Thirdly, the neural network model, to map the relationship between the key process parameters and defects, was constructed. The evaluation of the key process parameters enabled the empirical analysis of the shrinkage cavity and the evaluation of the loose defects of the formula. Finally, the sensitivity analysis of the impact of the key process parameters on the shrinkage cavity and loose defects was carried out, and the optimization window of the key process parameters was obtained.

### 2.2. Process Flow and Orthogonal Experiment

**Figure 2:**
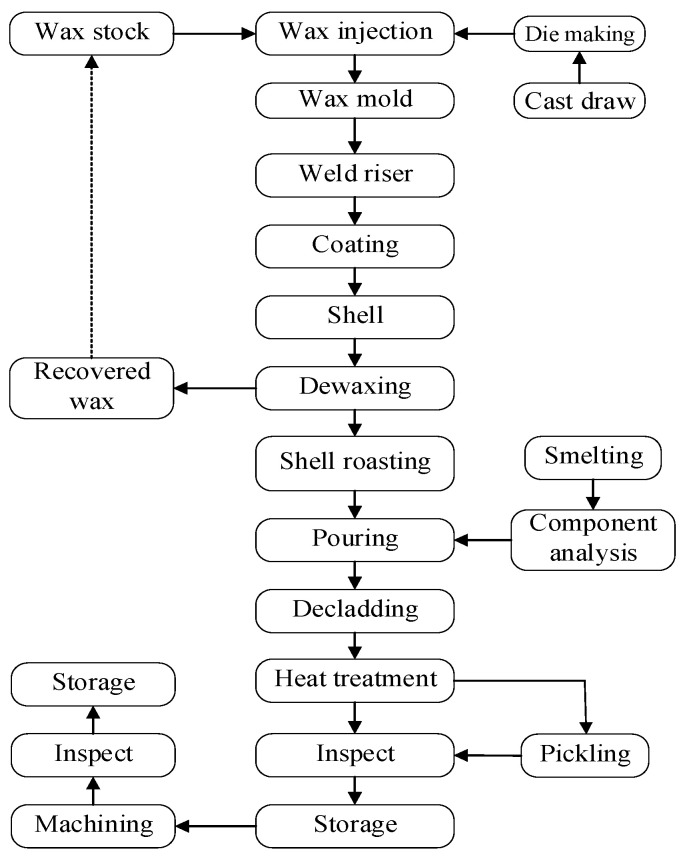
Casing process flow [29].

Ti alloy casing is typically a large and complex casting with a lengthy process flow. The main processes include wax mold pressing and combining, shell smelting and pouring, repair welding, pickling, hot isostatic pressing, X-ray inspection, and a machining final inspection, as shown in Figure 2. More than 70 processes affect the shrinkage cavity and loose defects of the casing. Defects result in the reduced strength of the casing. However, during smelting and pouring, the casing can revive different shrinkage cavities and loose defects. Thus, this paper discusses the effects of changes in the parameters of the smelting and pouring processes on the defects.

**Figure 3:**
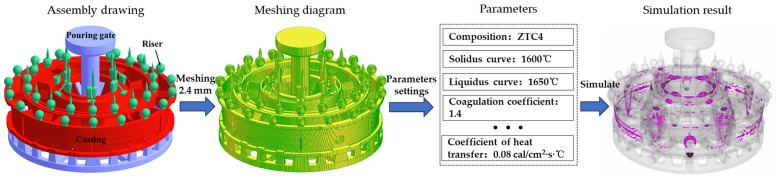
Simulation flow of shrinkage cavity formation and material loosening defects in a Ti alloy casing.

Among the casting methods are gravity casting, pressure casting, centrifugal casting, etc. We used InteCast CAE to simulate the gravity investment casting of the Ti-alloy casing. The overall simulation process of shrinkage cavity and loose defects in Ti-alloy casing is shown in Figure 3. In the casting process of the Ti-alloy casing, the casting process includes the casting, pouring gate, and the corresponding riser. The casing needs to be meshed in the simulation process. The thinnest part of the Ti alloy casing’s casting thickness was 2.419 mm. In order to ensure the connectivity of the casing’s thin-wall sections and the computing efficiency of the computer, the length, width, and height of the split grid are 2.4 mm. During the simulation, we set the composition and thermophysical properties of the ZTC4 Ti alloy casing, as shown in Table 1 and Table 2.

Ti alloy complex components filling and solidification are different. Pouring temperature, pouring time, shell temperature, and thickness are the main factors influencing the solidification process. To improve the accuracy and efficiency of the experiment, the solidification simulation’s key parameter ranges originated from the actual production process. In actual production, the key process parameters of some casings are abnormal, and the key process parameters are used after the abnormal casings are eliminated. The pouring temperature is set based on a smelting current ranging from 41 kA to 42 kA, a smelting duration of 14 min to 19 min, and a molten metal quality varying between 381 kg and 425 kg. The shell temperature is established based on an actual range of 271 °C to 340 °C. Similarly, the pouring time is set according to an actual duration, ranging from 5.7 s to 6.8 s. We set the levels and factors of the orthogonal experiment according to the distribution amplitude, frequency, and density interval of the process parameters. The range of pouring temperature, shell temperature, pouring time, and shell thickness are shown in Table 3. We set the shell thickness to 10 mm. The heat transfer coefficient from casting to air was set to 3347.2 J/m^2^·s·°C.

In order to investigate the role of pouring temperature, pouring time, shell temperature, and their interactions on the shrinkage cavity and loose defects of the casing casting, we reduced the number of simulated experiments, optimized the optimal procedure scheme, and provided reference criteria for various experimental scenarios at the level of random distributions. Therefore, we designed an orthogonal experiment [30,31,32,33] with three factors and four levels of pouring temperature, pouring time, and shell temperature, as shown in Table 4.

### 2.3. Construction of Neural Network Prediction Model

**Figure 4:**
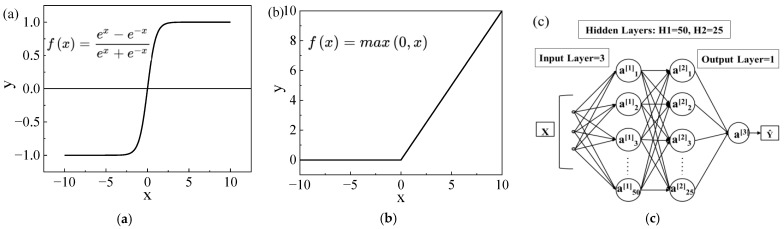
Neural network model. (**a**) Tanh. (**b**) ReLU. (**c**) Model structure.

Linear and nonlinear modeling of key process parameters and shrinkage defects, using a BP neural network, is established. The input characteristics are pouring temperature, pouring time, and shell temperature. The output results are shrinkage cavity number, shrinkage cavity volume, shrinkage loose number, and shrinkage loose volume, respectively. However, in the process of model construction, the activation function is the key for the neural network to be able to solve the nonlinear problem. At present, there are linear and nonlinear activation function. Tanh is used in the first hidden layer and ReLU is used in the second hidden layer as the activation function, the curves of which are shown in Figure 4a,b. We set the learning rate of training to 0.001, the number of iterations to 10,000. In terms of data division, due to the limited amount of data, the data are divided into 85% for the training set and 15% for the validation set. The model structure used is 3 × 50 × 25 × 1, as shown in Figure 4c.

To enhance the stability of the training outcomes, we implement normalization techniques to neutralize the disparities in dimensional characteristics among the key process parameters. In order to improve the accuracy of the model, we use the Bayesian regularization (BR) algorithm, which is a Bayesian-based method and can reduce the risk of overfitting and improve the generalization ability in the prediction model of the shrinkage number. For the prediction of the shrinkage volume, we use the Levenberg–Marquardt (LM) algorithm, which is better at predicting continuous variables. The loss function utilizes the mean squared error (MSE) to quantify the smallest discrepancy between the predicted and actual values. Additionally, to prevent overfitting, a strategy of randomly deactivating 20% of the neurons is employed.

## 3. Results and Discussion

### 3.1. Simulation Results Analysis

**Figure 5:**
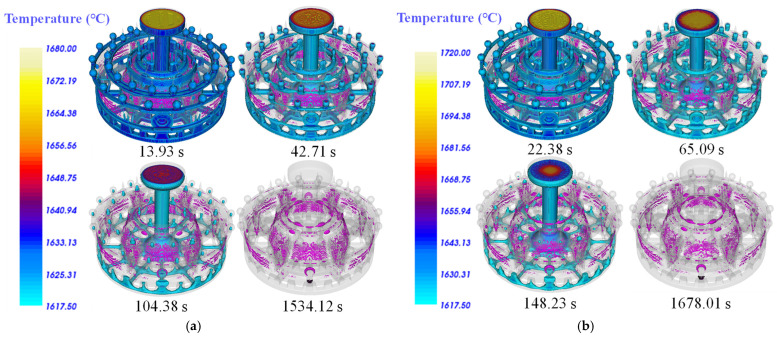
No.1 and No.5 solidification processes’ isolated liquid region, shrinkage cavity, and loose defects evolution, with solidification time. (**a**) No.1 sample. (**b**) No.5 sample.

Figure 5 shows the results of shrinkage evolution in the simulation of solidification processes No.1 and No.5. With the increase in solidification time, the defects are gradually presented in the isolated liquid phase region. In the comparison of the No.1 and No.5 solidification processes, it can be seen that the formation time of the porosity defects is different for different pouring temperatures and pouring times. In Ti alloy casing, larger and more shrinkage defects will occur with a higher pouring temperature and a longer cooling time. Hence, the shrinkage defects in the casing of No.5 are significantly higher than that of No.1.

**Figure 6:**
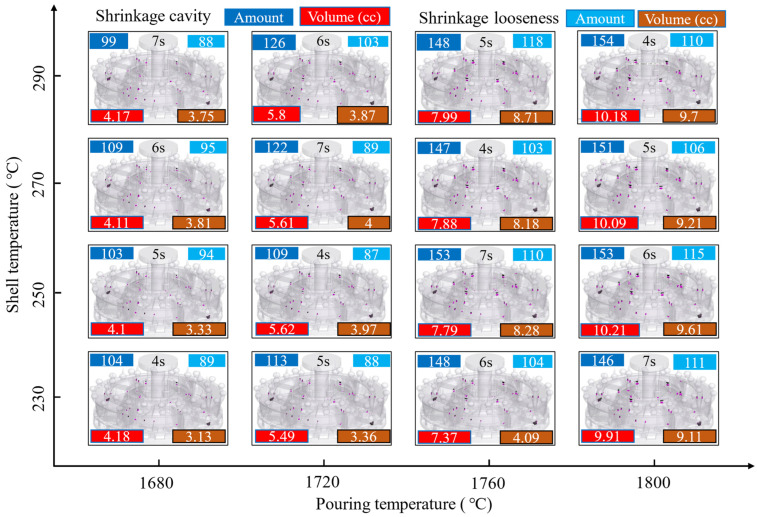
Influence of key process parameters on the number and total volume of shrinkage cavity and loosening.

Figure 6 shows the effect of pouring temperature, shell temperature, and pouring time on the number and volume of shrinkage cavities and loosening. In the three-dimensional diagram, shrinkage cavities are shown in red and shrinkage loosening is shown in black. Shrinkage cavity and shrinkage loosening are distinguished by a critical porosity boundary of 3%. The amount and volume are automatically counted by computer. The results show that the number and total volume of shrinkage cavity increase gradually with the increase in pouring temperature. In the conditions of shell temperatures of 230 °C and 250 °C, the number of shrinkage cavities increases sharply when the pouring temperature increases from 1720 °C to 1760 °C. This indicates that pouring temperature is the main factor affecting the number of shrinkage cavities. At shell temperatures of 250 °C, 270 °C, and 290 °C, a notable increase in total shrinkage loosening volume is observed, as the casting temperature rises from 1720 °C to 1760 °C. At a shell temperature of 230 °C, there is a marked increase in the total volume of shrinkage porosity when the pouring temperature is elevated from 1760 °C to 1800 °C. It indicates that pouring temperature should be controlled below 1720 °C to mitigate the total volume of shrinkage loosening, when the mold shell temperature exceeds 250 °C. At a pouring temperature of 1680 °C, the increase in the number and the total volume of shrinkage cavities and loosening is not significantly related to the increase in shell temperature. At a pouring temperature of 1760 °C, the total volume of shrinkage loosening increases significantly when the shell temperature is increased from 230 °C to 250 °C. This could be attributed to the attainment of a critical undercooling between the pouring temperature and the mold shell temperature.

In summary, to minimize the number and total volume of shrinkage cavities and porosity in the Ti alloy casing, it is imperative to regulate the pouring temperature to below 1720 °C. The liquidus line of the ZTC4 Ti alloy is lower than that of pure titanium, with a melting point of 1668 °C for pure titanium. In the actual casting process, it is inevitable that there will be heat transfer and solidification phenomena during the pouring and mold-filling stages, due to the flow of the molten metal. To counteract the adverse effects of premature solidification, which can lead to inadequate pouring, it is necessary for the titanium alloy melt to have a certain degree of superheat. The pouring temperature should be controlled at or above 1680 °C. The specific pouring temperature requires further investigation into the effects of casting process conditions, heat transfer conditions, shell thickness, shell material, and casting wall thickness on defect formation.

### 3.2. Correlation Analysis

**Figure 7:**
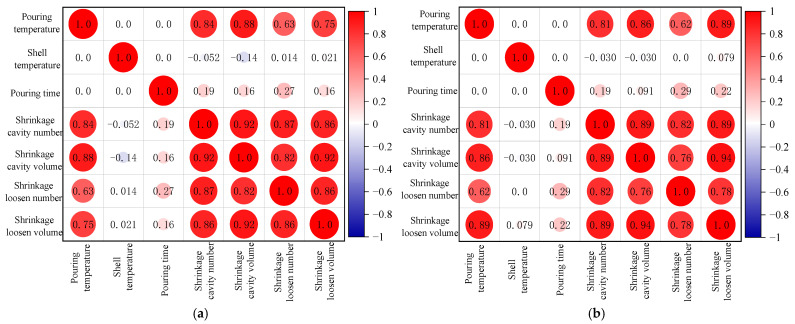
Correlation test of proposed parameters and simulation results. (**a**) Pearson relevance. (**b**) Spearman relevance.

The correlation between the simulated process parameters and shrinkage cavity and loose defects are analyzed, as shown in Figure 7. The results show that pouring temperature has a strong correlation with the number of shrinkage cavities, the total volume of shrinkage cavities, the number of shrinkage loosening, and the total volume of shrinkage loosening, according to the Pearson [34,35,36] and Spearman [37] correlation tests. In contrast, the mold shell temperature and pouring time do not exhibit a significant correlation with the simulation results, which is in agreement with the previous analytical findings.

### 3.3. Quantitative Multiple Regression Analysis of Pore Pine Defects

To establish a quantitative relationship between critical process parameters and shrinkage cavity and loose defects in casing, a sensitivity analysis was conducted using multiple regression analysis. The multiple regression analysis was conducted using a data set of 107 simulated experiments, which were generated through an orthogonal experimental design methodology. There are four prevalent methods for structural screening in multiple regression models: backward elimination [38], forward selection [39], principal components [40], and stepwise regression [41]. This paper uses the stepwise regression modeling approach.

**Figure 8:**
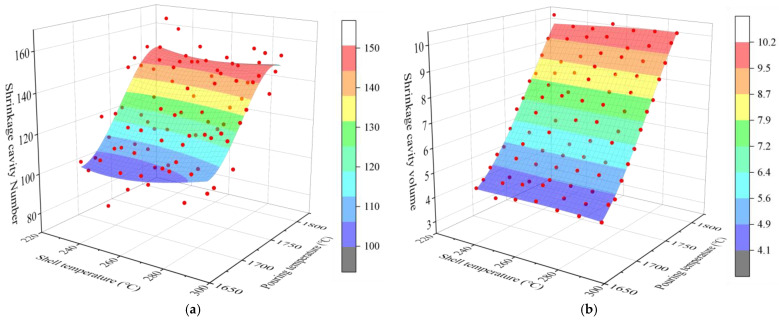
Regression model of shrinkage cavities’ number and volume with pouring temperature and shell temperature. (**a**) Shrinkage cavity number. (**b**) Shrinkage cavity volume.

**Figure 9:**
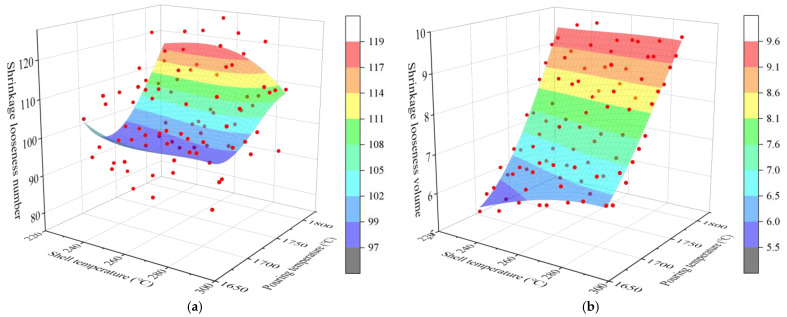
Regression model of shrinkage loosening number and volume with pouring temperature and shell temperature. (**a**) Count of shrinkage loosening. (**b**) Shrinkage loosening volume.

The optimal regression model for each defect is shown in Figure 8 and Figure 9. The influence of pouring and shell temperature on the defects establish a stepwise regression model with two key process parameters of mold shell temperature (X) and pouring temperature (Y) as independent variables. The pouring time was controlled to be a constant of 4 s. Taking the number of shrinkage cavities, the total volume of shrinkage cavities, the count of shrinkage loosening, and the total volume of shrinkage loosening as the fitted target, the stepwise regression models were established using the method of multiple regression analysis. We select the model with the best comprehensive performance for each performance index as the performance regression model, as shown in Table 5.

The multiple regression analysis examining the effects of pouring temperature and shell temperature on the formation of shrinkage cavities and loosening reveals that the adjusted R-squared (Adj.R^2^) value for the regression fit of shrinkage cavity volume is exceptionally high, at 99.4%. This indicates a strong and precise correlation between the volume of shrinkage cavities and both pouring and shell temperatures. Conversely, the Adj.R^2^ for the regression concerning the shrinkage loose number instances stands at 41.1%, indicating a considerably weaker fit. This suggests that the regression model for shrinkage loose number should be treated with caution and primarily used for reference purposes.

(1) $$\begin{array}{l} Z=340688-57*X+0.046*X^{2}-0.000036*X^{3}+0.052*XY-0.000008*X^{2}Y\\ \;\;\;\;\;\;-0.000014*XY^{2}-582*Y+0.332*Y^{2}-0.00006*Y^{3} \end{array}$$

(2) $$\begin{array}{l} Z=9574-2.45*X+0.001*X^{2}-0.0000013*X^{3}+0.0025*XY-0.000000025*X^{2}Y\\ \;\;\;\;\;\;-0.0000007*XY^{2}-16.05*Y+0.009*Y^{2}-0.0000017*Y^{3} \end{array}$$

(3) $$\begin{array}{l} Z=146526-202*X+0.172*X^{2}-0.000066*X^{3}+0.182*XY-0.000071*X^{2}Y\\ \;\;\;\;\;\;-0.000042*XY^{2}-220*Y+0.111*Y^{2}-0.000019*Y^{3} \end{array}$$

(4) $$\begin{array}{l} Z=4223-5.1*X-0.0018*X^{2}-0.0000014*X^{3}+0.0066*XY+0.0000016*X^{2}Y\\ \;\;\;\;\;\;-0.0000022*XY^{2}-6.4*Y+0.003*Y^{2}-0.00000047*Y^{3} \end{array}$$

In Equations (1) to (4), the quantitative relationship between the shrinkage defects and the pouring temperature, shell temperature and the two independent variables coupled can be seen. The coefficient corresponding to the pouring temperature is the largest, followed by mold temperature, which indicated that the shrinkage defects are the most sensitive to the pouring temperature, followed by mold temperature. Regarding the number and the volume of shrinkage cavities, the coefficient of the pouring temperature is considerably larger than the shell temperature, and more consideration is given to the effect of the pouring temperature on the shrinkage cavities. Regarding the count and the volume of shrinkage loosening, the coefficients of the pouring temperature and the shell temperature are closer to each other, and their sensitivities are also relatively close. The impact of the two key process parameters should be adequately considered and controlled.

### 3.4. Single-Factor Impact Law Analysis

Table 6 shows the key evaluation indexes of the shrinkage number and volume of the neural network training mode. The accuracy of the total volume of shrinkage cavities and the shrinkage loosening number are 99.9% and 73.9%, respectively. The overall accuracy of the training model using the neural network is higher than the multiple regression model. This is because neural networks can fit nonlinear functions, making their results more conducive to subsequent analysis.

**Figure 10:**
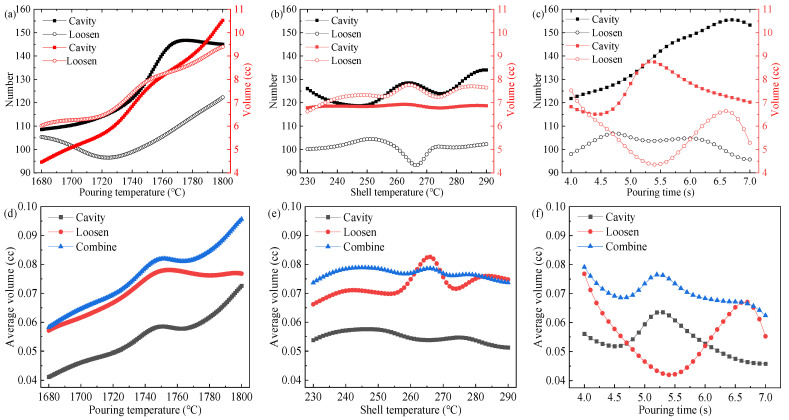
Relationship curves for the effect of key process parameters on shrinkage cavities and loose defects. (**a**) Count and volume of shrinkage cavity and loosening change with pouring temperature. (**b**) Count and volume of shrinkage cavity and loosening change with shell temperature. (**c**) Count and volume of shrinkage cavity and loosening change with pouring time. (**d**) Shrinkage cavity, shrinkage loosening, and combine average volume vary with pouring temperature. (**e**) Shrinkage cavity, shrinkage loosening, and combine average volume vary with shell temperature. (**f**) Shrinkage cavity, shrinkage loosening, and combine average volume vary with pouring time.

We train the model using a BP neural network, by controlling the change in a single key process parameter while setting the other process parameters to fixed values, as shown in Table 7. By controlling the input of key process parameters for the housing, the predicted values are obtained and corresponding relationship curves are plotted, as shown in Figure 10.

(5) $$\bar{\nu }=\frac{v}{n}$$

(6) $$v_{\mathrm{evaluate}}=v_{\mathrm{cavity}}+0.3\times v_{\mathrm{loosen}}$$

where $\bar{\nu }$ is the average volume of each shrinkage cavity or loose, cc, v is the total volume of shrinkage cavity or loose, cc, *n* is the number of shrinkage cavities or loosening, $v_{\mathrm{evaluate}}$ is the comprehensive evaluation volume, cc, $v_{\mathrm{cavity}}$ is the average volume of each shrinkage cavity, cc, and $v_{\mathrm{loosen}}$ is the average volume of each shrinkage loosening, cc.

In actual production, hot isostatic pressing (HIP) is conducted after the casting and solidification processes to increase the density of the casings. Therefore, when there are significant shrinkage cavities or loosening, the HIP process can markedly alter the dimensions of the casing, potentially leading to nonconforming products. To more intuitively and effectively evaluate the key process parameters affecting the casing defects, we select the composite shrinkage cavity and loosening volume as the evaluation index, as shown in Equation (5), given that shrinkage cavities typically inflict more damage than shrinkage loosening in casing castings and considering that shrinkage loosening is characterized by its scattered and fine cavities. Drawing on experience, this study quantifies 30% of the average volume of each shrinkage loosening occurrence as equivalent to the damage caused by shrinkage cavities. The established overall evaluation index for the average volume of each shrinkage cavity and loosening is shown in Equation (6).

The effect of pouring temperature on the number, total volume, and the average combined volume of shrinkage cavities and loosening are shown in Figure 10a,d. From Figure 10a, it can be observed that the number of shrinkage cavities increases slowly with the rise in pouring temperature. After reaching 1720 °C, the rate of increase in the number of shrinkage cavities accelerates, peaking at 145 at 1770 °C. Subsequently, at temperatures exceeding 1770 °C, a decline in the number of shrinkage cavities is observed. The count of shrinkage loosening initially diminishes as the casting temperature rises, reaching a minimum of 95 at 1725 °C. Subsequently, the number of shrinkage loosening starts to escalate. The number of shrinkage cavities and loosening both exhibit fluctuations. From Figure 10b, when converted to the combined volume per shrinkage defect, the combined volume trend increases with the increase in pouring temperature. The volume decreases between 1750 °C and 1765 °C, which correlates with the sharp increase in the number of shrinkage cavities. In summary, in order to minimize the average combined volume of each shrinkage, we should endeavor, as much as possible, to maintain the casting temperature at 1680 to 1700 °C.

The effects of the shell temperature on the number, total volume, and the average combined volume of shrinkage cavities and loosening are shown in Figure 10b,e. The number of shrinkage cavities fluctuates with the increase in mold shell temperature. The lowest number of shrinkage cavities was achieved at 247 °C. The lowest count of shrinkage loosening was achieved at 267 °C. The total volume of shrinkage cavities did not change significantly with the increase in shell temperature. The minimum total volume of shrinkage loosening was achieved at 230 °C. The effect of shell temperature changes on the average combined volume is relatively small. The average combined volume is the lowest at temperatures ranging from 230 to 235 °C and from 270 to 290 °C. To facilitate control of the shell temperature, it should be maintained between 270 to 290 °C.

The effect of pouring time on the number, total volume, and the average combined volume of the defects, are shown in Figure 10c,f. The number of shrinkage cavities increases with the increase in pouring time and decreases after 6.75 s. The number of shrinkage loosening is maximized at 4.5 s. The total volume of shrinkage cavity is minimized at 4.5 s. Later, it increases to a maximum of 8.8 cc with the pouring time. The total volume of shrinkage loosening decreases with casting time and reaches a minimum of 4.2 cc at 5.4 s. Subsequently, it increases and then decreases again as casting time extends. From Figure 10f, the pouring time should be maintained within the range of 5.8 to 7 s.

The influence of single factor variation range on the defects was shown in Table 8. The pouring temperature has the greatest influence on the number and the total volume of the shrinkage defects. In addition, the pouring time has a more significant effect on the shrinkage defects than shell temperature. The results show that the effects of the key process parameters on shrinkage defects, in a descending order of sensitivity, are as follows: the pouring temperature, the pouring time, and the shell temperature.

### 3.5. Multi-Factor Influence Law Analysis

**Figure 11:**
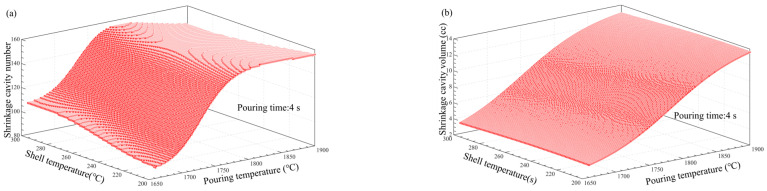
Effect of pouring and shell temperature on the number and total volume of shrinkage cavities. (**a**) Effect of pouring temperature and shell temperature on the number of shrinkage cavities. (**b**) Effect of pouring temperature and shell temperature on the volume of shrinkage cavities.

**Figure 12:**
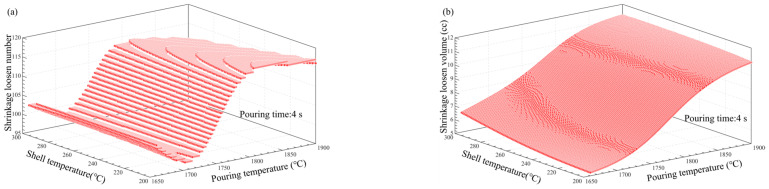
Effect of pouring and shell temperature on the number and total volume of shrinkage loosening. (**a**) Effect of pouring temperature and shell temperature on the number of shrinkage loosening. (**b**) Effect of pouring temperature and shell temperature on the volume of shrinkage loosening.

**Figure 13:**
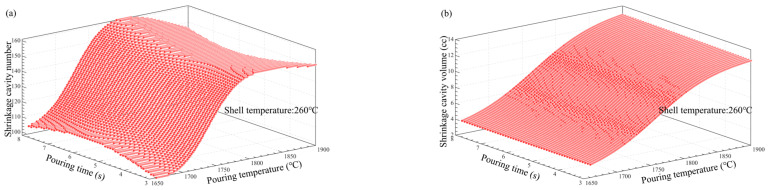
Effect of pouring temperature and time on the number and total volume of shrinkage cavities. (**a**) Effect of pouring temperature and pouring time on the number of shrinkage cavities. (**b**) Effect of pouring temperature and pouring time on the volume of shrinkage cavities.

**Figure 14:**
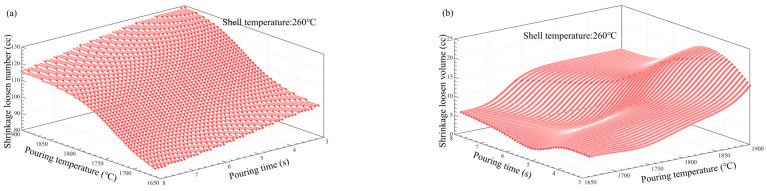
Effect of pouring temperature and time on the number and total volume of shrinkage loosening. (**a**) Effect of pouring temperature and pouring time on the number of shrinkage loosening. (**b**) Effect of pouring temperature and pouring time on the volume of shrinkage loosening.

**Figure 15:**
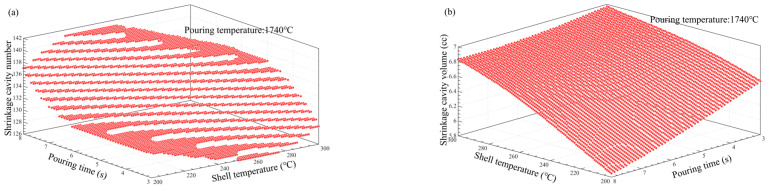
Effect of shell temperature and pouring time on the number and total volume of shrinkage cavities. (**a**) Effect of pouring time and shell temperature on the number of shrinkage cavities. (**b**) Effect of pouring time and shell temperature on the volume of shrinkage cavities.

**Figure 16:**
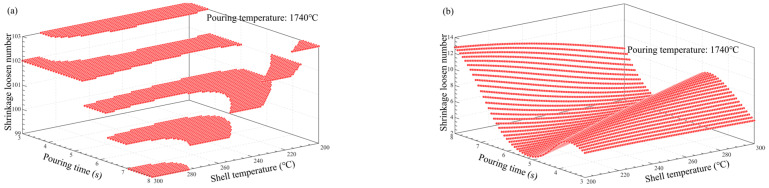
Effect of shell temperature and pouring time on the number and total volume of shrinkage loosening. (**a**) Effect of pouring time and shell temperature on the number of shrinkage loosening. (**b**) Effect of pouring time and shell temperature on the volume of shrinkage loosening.

Figure 11, Figure 12, Figure 13, Figure 14, Figure 14, Figure 15 and Figure 16 depict the application of a two-factor analysis to assess how key process parameters influence the variability of hole loosening defects. Figure 11 and Figure 12 show the effect of pouring temperature and shell temperature on the shrinkage defects. Figure 13 and Figure 14 show the effect of pouring temperature and time on the shrinkage defects. Figure 15 and Figure 16 show the effect of pouring time and shell temperature on the shrinkage defects. The figures demonstrate that there is an interaction between the two influencing factors on the shrinkage defects. The greatest impact is from the pouring temperature, followed by the pouring time, with the shell temperature having the least effect. The results show that, when the pouring temperature is 1650 °C, the total volume of shrinkage cavities and loosening is small, according to the neural network prediction, due to the cooling that occurs during the filling process, which can lead to casting defects. It is necessary to control the pouring temperature within the range of 1680 to 1700 °C.

In summary, the established BP neural network model accurately predicts the influence of key process parameters variations on the shrinkage cavities and loose defects, as shown in Figure 11, Figure 12, Figure 13, Figure 14, Figure 15 and Figure 16. A set of key process parameter windows and fluctuation control strategies have been established by combining this with multiple regression analysis. The original process parameter control ranges and the optimized process parameter ranges are shown in Table 9.

### 3.6. Comparative Analysis of Actual Production and Analysis Results

**Figure 17:**
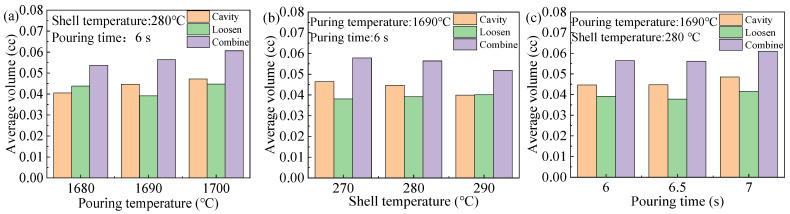
The relationship between key process parameters and average volume after optimization. (**a**) Effect of pouring temperature on the average volume. (**b**) Effect of shell temperature on the average volume. (**c**) Effect of pouring time on the average volume.

After analysis of the results of the constructed neural network prediction model and evaluation formula, selections within the process optimization scope were made for simulation validation, as shown in Figure 17. The results show that the optimized range produced fewer shrinkage cavity and loose defects than the unoptimized range. Simulation validation demonstrates that the results obtained from using the neural network model to predict shrinkage cavity and loose defects were reliable and can accurately identify the optimal range of key process parameter values.

Based on the simulation results, the outcomes from the analysis using multiple regression and neural network models were compared with the actual production range of key process parameters for the Ti alloy casing by a certain research institute, as shown in Table 10. When using a water-cooled copper crucible for vacuum melting, only the smelting current, time, and the amount of smelt metal can be measured. The comparison of results shows that the optimized process window is within the actual production process range. The constructed neural network prediction model and evaluation formula meet the production requirements of the enterprise and can provide a reference for reducing the occurrence of shrinkage defects in the casting of casings.

**Figure 18:**
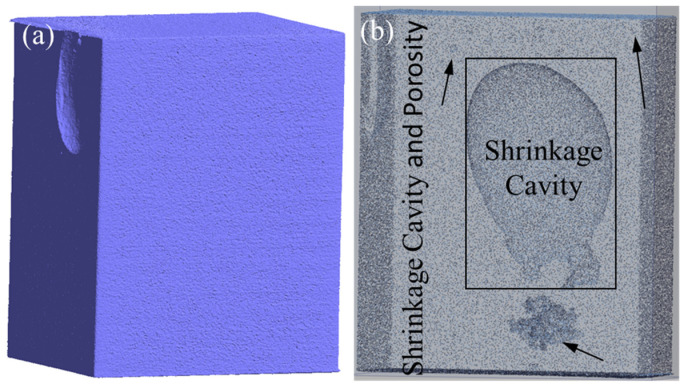
Shrinkage cavity and loosening of pouring channel. (**a**) Appearance of pouring channel. (**b**) Internal of pouring channel.

**Figure 19:**
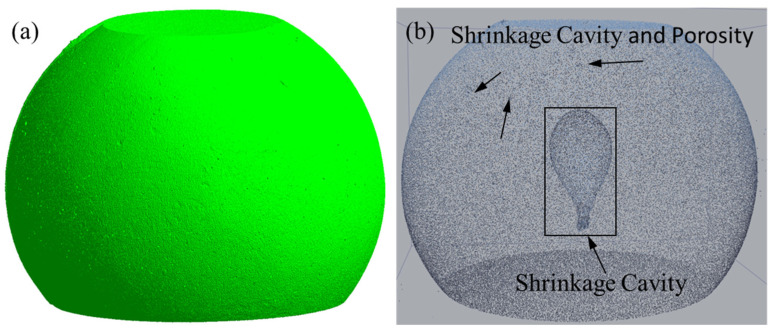
Shrinkage cavity and loosening of riser. (**a**) Appearance of riser. (**b**) Internal of riser.

In industry, the process within the optimization window is compared with the results outside the range, using a casting channel for casing with pouring temperature of 1708 °C, a shell temperature of 298 °C, and a pouring time of 6.2 s, as shown in Figure 18. A piece of the casting channel is cut for testing by the CT (Computed Tomography) statistics; the volume of the casting channel is 10,954.08 mm^3^, the volume of pores is 131.36 mm^3^, and the porosity is 1.199%. Another process parameter is within the optimization window, with a pouring temperature of 1698 °C, a shell temperature of 288 °C, and a pouring time of 6.5 s, as shown in Figure 19. The riser has a volume of 26,584.07 mm^3^, the volume of pores is 249.58 mm^3^, and the porosity is 0.94%. When comparing the proportion of porosity defects for the two process parameters, there are fewer defects in the optimization window range, which indicated that the use of numerical simulation and neural networks in tandem to find the optimization window has significant results.

## 4. Conclusions

In this study, the data set of complex castings of Ti alloy casings was constructed by undertaking an orthogonal simulation experiment. Based on the data set, multiple regression and BP neural network models were used for quantitative analysis, to find the fluctuation window of the optimized process parameters. The analysis conclusions are as follows:

(1) The orthogonal experimental simulations conducted indicated that, for ZTC4 Ti alloy casings, the occurrence of shrinkage defects is reduced when the temperature is maintained between 1680 and 1720 °C. Furthermore, correlation analysis shows that both the number and the volume of shrinkage cavity and loosening are influenced by the pouring temperature. However, the effect of shell temperature and pouring time on the defects is not significant.
(2) The multiple regression prediction model quantitatively described the impact of pouring temperature and shell temperature on the shrinkage cavity and loose defects. The fitting accuracy for the total volume of shrinkage cavities reached 99.5%. In the fitting of the number and volume of shrinkage cavities, shrinkage cavities were more sensitive to the pouring temperature. In the fitting of the number and volume of loose defects, the sensitivity of loose defects to the two key process parameters was similar, which indicated that the influence of both should be considered simultaneously.
(3) Based on the neural network prediction model and the comprehensive evaluation formula for shrinkage cavity and loose defects, the sensitivity of the casing defects to key process parameters was highest for pouring temperature, then pouring time, and lowest for shell temperature. Additionally, the key process parameter windows were optimized: pouring temperature at 1680–1700 °C, shell temperature at 270–290 °C, and pouring time at 5.8–7 s.
(4) By integrating numerical simulation with neural network methodologies, this study effectively addresses the previously laborious process of determining optimal windows for key process parameters through simulation alone. Furthermore, it delineates the distinct advantages of neural network analysis over traditional multivariate regression. While multivariate regression can provide sensitivity coefficients that link porosity defects in casings to key process parameters, its precision for the number of shrinkages and loose defects is notably insufficient. In contrast, the BP neural network prediction model demonstrates superior accuracy, relative to multivariate regression, offering a significant improvement in the precision of the number of shrinkages and loose defects in casings.

## Figures and Tables

**Table 1:** ZTC4 Ti alloy composition content in terms of weight percent (wt%).

Elements	Ti	Al	Fe	H	Si	V	C	O	N
Content	89.3	6.2	0.2	0.01	0.1	4	0.06	0.1	0.03

**Table 2:** ZTC4 Ti alloy thermophysical properties.

Thermal Emissivity (W/m^2^)	Latent Heat (J/g)	Liquidus (°C)	Solidus (°C)	Critical Solid Fraction	Solidification Coefficient (mm/s^1/2^)	Shrinkage of Phase Transition	Liquid Shrinkage Degree
0.13	335.62	1650	1600	0.65	1.4	0.04476	2.2102 × 10^−5^

**Table 3:** Range of values for simulated process parameters.

Pouring Temperature (°C)	Pouring Time (s)	Shell Temperature (°C)	Shell Thickness (mm)	Heat Transfer Coefficient (J/m^2^·s·°C)
1680–1800	4–7	230–290	10	3347.2

**Table 4:** L_16_(4^3^) orthogonal table design.

No.	Pouring Temperature (°C)	Shell Temperature (°C)	Pouring Time (s)	No.	Pouring Temperature (°C)	Shell Temperature (°C)	Pouring Time (s)
1	1680 (1)	230 (1)	4 (1)	9	1760 (3)	230 (1)	6 (3)
2	1680 (1)	250 (2)	5 (2)	10	1760 (3)	250 (2)	7 (4)
3	1680 (1)	270 (3)	6 (3)	11	1760 (3)	270 (3)	4 (1)
4	1680 (1)	290 (4)	7 (4)	12	1760 (3)	290 (4)	5 (2)
5	1720 (2)	230 (1)	5 (2)	13	1800 (4)	230 (1)	7 (4)
6	1720 (2)	250 (2)	4 (1)	14	1800 (4)	250 (2)	6 (3)
7	1720 (2)	270 (3)	7 (4)	15	1800 (4)	270 (3)	5 (2)
8	1720 (2)	290 (4)	6 (3)	16	1800 (4)	290 (4)	4 (1)

**Table 5:** Evaluation for regression modeling of shrinkage cavity and loosening number and volume.

Coefficient of Determination	Shrinkage Cavity Number	Shrinkage Cavity Volume	Shrinkage Loose Number	Shrinkage Loose Volume
R^2^	0.827	0.995	0.471	0.941
Adj.R^2^	0.808	0.994	0.411	0.934

**Table 6:** Evaluation for neural network model of casing shrinkage number and volume.

Evaluation	Shrinkage Cavity Number	Shrinkage Cavity Volume	Shrinkage Loose Number	Shrinkage Loose Volume
Training R^2^	0.936	0.999	0.716	0.961
Validation R^2^	0.920	0.999	0.739	0.955
MSE	6.281	0.10	6.26	0.766

**Table 7:** Single-factor process parameter control variables.

Pouring Temperature (°C)	Shell Temperature (°C)	Pouring Time (s)
1680–1800	260	4
1740	230–290	4
1740	260	4–7

**Table 8:** Range of variation in shrinkage cavity and loose defects in one-factor prediction experiments.

Factors	Range	Cavity Number	Cavity Volume (cc)	Loosening Number	Loosening Volume (cc)
Pouring temperature (°C)	1680–1800	109–147	4.5–10.5	97–123	6–9.4
Shell temperature (°C)	230–290	119–134	6.8–6.9	93–105	6.6–7.8
Pouring time (°C)	4–7	122–156	4.5–8.8	96–107	4.4–7.5

**Table 9:** Optimization window for key process parameters.

Parameters	Original Range	Optimization Range
Pouring temperature (°C)	1680–1800	1680–1700
Shell temperature (°C)	230–290	270–290
Pouring time (s)	4–7	5.8–7

**Table 10:** Actual vs. predicted process parameter ranges.

Actual and Predicted Range	Parameters	Parameter Ranges
Actual	Smelting current (kA), smelting time (min), smelting metal (kg)	41–42, 14–19, 381–425
	Shell temperature (°C)	271–340
	Pouring time (s)	5.7–6.8
Optimization window	Pouring temperature (°C)	1680–1700
	Shell temperature (°C)	270–290
	Pouring time (s)	5.8–7

## Data Availability

Data are contained within the article.
